# A Possible Role of Akkermansia muciniphila in the Treatment of Olanzapine-Induced Weight Gain

**DOI:** 10.7759/cureus.55733

**Published:** 2024-03-07

**Authors:** Francesca Bertossi

**Affiliations:** 1 Department of Mental Health, Azienda Sanitaria Universitaria Giuliano Isontina, Trieste, ITA

**Keywords:** antipsychotic-induced weight gain, gut-brain axis, inflammation, metabolic syndrome, olanzapine, akkermansia muciniphila, gut microbiota

## Abstract

Second-generation antipsychotics are mainly used in both acute and long-term treatment of major psychiatric disorders. Although better tolerated than first-generation antipsychotic drugs, they can frequently induce weight gain and metabolic disorders, of these, olanzapine is one of the drugs more likely to induce these side effects. There is consistent evidence of the role of gut microbiota in modulating the gut-brain axis with complex crosstalk with the host involving satiety signaling pathways, food intake behavior, and weight and metabolic regulation. Second-generation antipsychotics induce important gut microbiota modification thus contributing together with the central and peripheral receptors blockade mechanism to weight gain induction and metabolic impairment. These drugs can alter the composition of gut microbiota and induce dysbiosis, often reducing the concentration of *Akkermansia muciniphila*, a bacterium that is also decreased in patients with diabetes, obesity, metabolic syndrome, or chronic inflammatory diseases. Probiotic administration can be a safe and well-tolerated approach to modulate microbiota and offer an integrative strategy in psychiatric patients suffering antipsychotic side effects.

Multiple strain probiotics and *Akkermansia muciniphila *alone have been administered both in mice models and in clinical populations demonstrating efficacy on antipsychotic-induced metabolic impairment and showing a contribution in reducing induced weight gain. *Akkermansia muciniphila* can improve several parameters altered by olanzapine administration, such as weight gain, insulin resistance, hyperglycemia, liver function, systemic inflammation, and gut barrier function. Although we do not have jet trials in the psychiatric population, this probiotic may be a complementary approach to treating olanzapine-induced weight gain and metabolic side effects.

## Introduction and background

Second-generation antipsychotics (SGAs), such as olanzapine, risperidone, quetiapine, and aripiprazole, are mainly used in both the acute and long-term treatment of major psychiatric disorders. Although SGAs are less likely to induce movement disorders and are effective in treating depressive symptoms compared to first-generation antipsychotics, they have a high metabolic burden and can induce weight gain [1,2]. Antipsychotic-associated weight gain begins during the first weeks of treatment and may continue over long-term treatment with some SGAs, for this reason, it is recommended to monitor weight, waist circumference, and blood tests over time [1].

Psychiatric patients have a reduction of life expectancy of 10-20 years compared to the general population [3,4], one-third of them suffer from metabolic syndrome [5] and have three times the risk of developing cardiovascular diseases, including those that are potentially fatal [4,6]. As a consequence, it is of primary importance to prevent any risk factor wherever possible. Metabolic side effects and induced weight gain can also negatively impact compliance, increasing the risk of disease relapse, moreover, these side effects are difficult to manage in this population with the specific strategies commonly used [7-9].

Gut microbiota is involved in several physiological processes by modulating the gut-brain axis with complex crosstalk with the host involving satiety signaling pathways, food intake behavior, and weight and metabolic regulation [10,11]. Gut microbiota can contribute to host metabolism through different mechanisms such as altering endocrine and satiety signaling, altering the capacity for energy harvest, influencing the gut and systemic inflammation mechanism, and modifying lipidic metabolism modulation [11-13].

SGAs induce important gut microbiota modification contributing together with the pharmacological receptors blockade mechanism to weight gain induction and metabolic impairment [14]. These drugs can alter the composition of gut microbiota and induce dysbiosis, often reducing the concentration of *Akkermansia muciniphila*, a bacterium that is also decreased in patients with diabetes, obesity, metabolic syndrome, or chronic inflammatory diseases [15].

This narrative review aimed to explore the mechanism of antipsychotic-induced weight gain and metabolic impairment focusing on the role of microbiota modifications in order to elucidate the rationale of the supplementation with probiotics as an adjunctive and physiologically based strategy to treat SGAs' side effects. As olanzapine is one of the SGAs mostly involved in weight gain and metabolic impairment, this review focuses on this compound. It examines the existing literature on probiotic implementation, with a particular focus on *Akkermansia muciniphila*, which has been proven effective in treating metabolic syndrome and weight imbalance in both animal and human studies [16,17].

In the current literature, there are two randomized control trials (RCTs) on mice models administered with olanzapine, the first with VSL#3, the second with *Akkermansia muciniphila,* and five heterogeneous RCTs on psychiatric patients treated with diverse antipsychotics and administered with different types of probiotics alone or in combination with dietary fiber or vitamin D with the aim to evaluate weight reduction and metabolic profiles.

## Review

Mechanism of induced weight gain and metabolic side effects

Although not completely understood, SGAs can induce weight gain both through the central and peripheral receptor blockade mechanisms and by gut microbiota modifications [18,19]. The central 5HT2C, H1, and D2 receptor blockade can induce both altered satiety and reward signaling that can increase food intake and influence energy expenditure [20,21]. The D2 presynaptic blockade, especially in the mesolimbic area, can alter the normal reward pathway, inducing the need to increase food intake to transmit satisfaction signaling [22].

Olanzapine has multiple profiles of receptor antagonism including dopamine D1, D2, D3, and D4 receptors, serotonin 5HT2A, 5HT2C, 5HT3, and 5HT6 receptors, alpha-1 adrenergic receptor, histamine receptor H1, and multiple muscarinic receptors, this compound is one of the SGAs that more likely can cause weight gain and metabolic side effects [1]. Olanzapine-induced weight gain is due to the central 5HT2C receptor, whose blockade can cause hyperphagia in rodents, and to the central H1 receptor and M3 receptor blockade [23-25].

The long-term hypothalamic H1 receptor blockade increases appetite and consequently food intake and decreases energy expenditure through thermogenesis inhibition, while the olanzapine peripheral M3 receptor on pancreas beta cells can decrease insulin secretion, exposing the patient to risk of metabolic syndrome [25-29]. Moreover, olanzapine activates hepatic gluconeogenesis inducing glucose-6-phosphatase (G6P-ase) mRNA and neuropeptide Y (NPY) and agouti-related peptide (AgRP) concentration levels, which are central orexigenic peptides [30-32].

SGAs can reduce anorexigenic hormones, for example, a reduction of circulating glucagon-like peptide-1 (GLP-1) induced by olanzapine, clozapine, and quetiapine can alter both glucose homeostasis and taste perception, in particular sweet taste perception causing carbohydrate and highly caloric food cravings [33].

The role of gut microbiota in weight gain

There is consistent evidence concerning the role of gut microbiota in balanced weight, food intake, and metabolism regulation, thus playing a pivotal role in the gut-brain axis [10]. Obesity is always associated with dysbiosis with an altered Firmicutes/Bacteroidetes ratio and alpha and beta diversity reduction, there is some experimental evidence that obesity is a transmissible tract via fecal microbiota transplantation [34]. Gut microbiota can contribute to host metabolism through different mechanisms [11], such as altering endocrine and satiety signaling [12,28], increasing the capacity for energy harvest [13], modifying lipidic metabolism modulation [35], and influencing the gut and systemic inflammation mechanism [36].

Short-chain fatty acids (SCFAs) produced only by certain microbiota bacteria play a central role in several host functions due to their anti-inflammatory and immunomodulation properties, neuroimmune regulation, epigenetic modifications, and appetite signaling [12]. SCFAs via FFARs (free fatty acid receptors) binding can induce the release of anorexigenic peripheral hormones (PYY, GLP-1) that can modify insulin, leptin, and ghrelin concentration, though they can also influence hypothalamic release of pro-opiomelanocortin (POMC) and cocaine-and-amphetamine-regulated transcript (CART), orexigenic neuropeptide Y (PNY), and agouti-related peptide (AgRP) that can ultimately modify eating behavior and the related dimensions of cognition, impulsivity, mood and anxiety [12].

The administration of SCFAs in obese mice with a free diet can reduce weight and improve triglycerides, fasting insulin, and leptin concentrations [37,38]; weight loss has also been observed in obese humans after SCFA supplementation [39]. Certain probiotic strains have an indirect antiobesity activity by reversing the source of proinflammatory stimuli linked with low-grade endotoxemia and thus affecting the inflammatory response [40]. The cross-talk between gut microbiota and its metabolites and the host can play a central role in maintaining the mucosal barrier, which is demonstrated to have been altered in obese subjects [41].

Modifications of SGAs on gut microbiota

Gut microbiota is involved in the regulation of every physiological host process and participates in xenobiotic and drug metabolism, often participating in their therapeutic effect; however, at times drug microbiota modification can contribute to some side effect manifestations [42]. Psychotropic drugs can influence gut microbiota compositions, and in some cases, they can exert antimicrobial properties [14,43].

SGAs can modify the microbiota composition altering the Firmicutes/Bacteroidetes ratio and reducing species linked to a lean microbiota, such as *Akkermansia muciniphila* and Alistipes. The concentration of *A. muciniphila* is reduced in patients with bipolar disorder treated with SGAs [44] and in mice administered with olanzapine [45], modifications of gut microbiota with olanzapine have also been demonstrated to have been altered in other studies [46,47]. These drugs through a complex mechanism of signaling involving microbiota can impair glucose and lipidic metabolism, induce inflammatory pathway modifications, and increase energy harvest from food [13,48].

Risperidone, for example, through microbiota dysregulation can induce a 16% reduction in total resting metabolic rate (RMR) in mice, the equivalent effect on humans on a 2000 kcal/day diet, a 16% reduction would be equivalent to consuming a cheeseburger every day, thus inducing an obesogenic trend [49]. The reduction of the resting metabolic rate can also be observed after microbiota transplantation in drug-naive mice, proving the central role of microbiota-induced modification by SGAs in inducing weight gain [49].

Strong evidence of the role of altered microbiota in weight gain induction is the administration of antibiotic therapy together with SGAs to prevent or reverse weight gain, although this is unsuitable as a strategy for treating this side effect due to the well-known risk of antibiotic resistance [46]. Olanzapine is often associated with serious metabolic side effects including weight gain and increased visceral fat, in the long term it has been demonstrated that this alters the composition of gut microbiota in rats [47,46]. A combined antibiotic therapy coadministered with olanzapine in rats attenuated body weight gain, visceral fat deposition, macrophage infiltration of adipose tissue, and plasma-free fatty acid levels, all of which were increased by olanzapine alone [46].

Olanzapine, like other antipsychotics, can cause metabolic dysregulation independently of its effect on increasing body weight and visceral fat mass, consequently exposing these patients to the risk of developing metabolic diseases. Metabolic dysregulation has been seen in patients after long-term olanzapine treatment in the absence of overt weight gain in both clinical and preclinical studies [50-52].

Management of antipsychotic-induced weight gain

Several strategies are used to treat SGAs-induced weight gain ranging from lifestyle intervention and advice to switching to another antipsychotic with a reduced propensity to induce metabolic adverse effects, or proposing augmentation with another medication when switching is not possible [18,32]. Among pharmacological intervention strategies, there is augmentation with metformin, bupropion, topiramate, amantadine, naltrexone, and samidorphan, the latter recently approved by the FDA and sold combined with olanzapine [53,18].

Lifestyle interventions, such as exercise, nutrition interventions, psychoeducational programs, or cognitive behavioral therapy (CBT), have shown a modest efficacy in reducing weight or treating metabolic syndrome and they need to be well structured to be proposed to psychiatric patients who often have a poor and sedentary lifestyle and are desocialized [54-56].

It is interesting to note that several interventions proposed to reduce SGAs-induced weight gain are associated with microbiota modification or dysbiosis treatment. For example, metformin exerts a regulating action on gut microbiota, improving *Akkermansia muciniphila* and SCFAs-producing bacteria, such as Butyrivibrio, *Bifidobacterium bifidum*,and Megasphaera, while diet and exercise are among the most effective intervention for gut microbiota regulation [57-59].

Probiotic supplementation has been studied as a treatment for weight gain and metabolic syndrome in both animal and human studies [60,61], and efficacy has been found in trials with *Lactobacillus casei*, *Lactobacillus rhamnosus*, *Lactobacillus gasseri*, *Lactobacillus plantarum*, *Bifidobacterium infantis*, *Bifidobacterium longum*, and *Bifidobacterium breve* [62,63]. A four-month implementation with VSL#3 (*Streptococcus thermophilus* DSM24731, *L. acidophilus* DSM24735, *L. delbrueckii subsp. bulgaricus* DSM24724, *L. paracasei* DSM24733, *L. plantarum* DSM24730, *B. longum *DSM24736, *B. infantis *DSM24737, and *B. breve *DSM24732) in obese children, lead to a reduction of BMI, increase of GLP-1, and reduction of steatohepatitis compared to controls [64]. An eight-week case-control study conducted on obese adults with a symbiotic administration (*Lactobacillus acidophilus*, *Lactobacillus casei*, *Bifidobacterium bifidum*, and inulin) demonstrated a decrease in weight, cholesterol, and triglycerides [65].

VSL#3 probiotic was administered to olanzapine-treated female mice for four weeks with statistically significant attenuation of drug-induced body weight gain, uterine fat deposition, impaired glucose tolerance, and insulin resistance [45].

On a clinical population with olanzapine-induced weight gain diverse probiotics have been tested in two RCTs, one study was conducted on patients suffering the first episode of psychosis and the other on subjects with a longer clinical history.

Two randomized clinical trials were conducted on first-episode schizophrenia patients who were randomized to receive in the first study either olanzapine plus multiple strain probiotics Bifico (Bifidobacterium, Lactobacillus, and Enterococcus) or olanzapine monotherapy for 12 weeks, and in the second study to be administered with either olanzapine plus probiotics and dietary fiber or olanzapine monotherapy for 12 weeks, all patients had no medical comorbidities [66]. No significant differences in weight gain were observed in probiotic-administered patients but when given together with fiber a better outcome in weight loss was reached, though not statistically significant. The insulin resistance index (IRI) was significantly improved in the probiotic-administered group (p=0.005) and when given together with dietary fiber the outcome was even better (p<0.001). Despite the limits due to the short time of the trial, the two studies emphasize the positive role of modulating gut microbiota in reverting insulin resistance induced by olanzapine and possibly influencing the weight balance over a longer time.

In a 12-week clinical trial, 70 patients diagnosed with schizophrenia or schizoaffective disorder were randomly assigned either to receive olanzapine alone or olanzapine plus a Bifidobacterium species probiotic. The differences between the two groups in weight change (2.4 kg versus 1.1 kg, p<0.05) and BMI (0.9 versus 0.4, p<0.05) were seen only in the first four weeks of treatment, while they were non-significant in the following weeks, highlighting the importance of an early intervention and suggesting the necessity to conduct long term studies [67].

Three studies with multiple strained probiotic administrations alone or in combination with fiber or vitamin D were conducted on a clinical population with long-term antipsychotic treatment. In an RCT study, 100 patients with a diagnosis of schizophrenia or bipolar disorder who had significant weight gain after the administration of SGAs were randomly assigned on a 12-week trial to multiple strain probiotics Bifico alone or combined with fiber versus placebo [68]. The patients were maintained with the same SGAs and subjects with comorbidities were excluded. Despite the limits due to the choice of selecting patients with different SGAs and the heterogeneity of the clinical population in the sample, this clinical study showed the effectiveness and safety of dietary fiber and probiotics used alone or in combination to mitigate atypical antipsychotic-induced metabolic side effects and weight gain.

Better results were obtained in another study in which 136 patients with a diagnosis of schizophrenia or bipolar disorder in SGAs treatment were randomized to four treatment groups comparing Bifico probiotic and dietary fiber alone or in combination and a placebo [69]. The trial lasted 12 weeks, patients with comorbidities were excluded and those enrolled continued to receive their respective atypical antipsychotic medications or mood stabilizer drugs without change throughout the study. The study demonstrated that patients receiving probiotics plus dietary fiber had a 2.36 kg decrease in weight compared with a 2.63 kg increase in weight in the placebo group, the difference was significant, the insulin resistance and the cholesterol level significantly improved in the treatment group versus the placebo group. In this study, microbiota composition was studied at the beginning and end of the treatment performing 16S ribosomal RNA sequencing on stool samples, demonstrating the increase of microbiota diversity and richness in the symbiotic treatment group that was associated with favourable weight loss.

A 12-week RCT was conducted on 60 patients with chronic schizophrenia to receive either 50,000 IU vitamin D3 every two weeks plus daily LactoCare probiotic containing *Lactobacillus acidophilus*, *Bifidobacterium bifidum*, *Lactobacillus reuteri*, and *Lactobacillus fermentum* or a placebo to evaluate the impact on clinical symptoms and metabolic profile [70]. The treatment group showed a highly significant decrease in fasting plasma glucose, serum insulin concentrations, homeostasis model assessment-estimated insulin resistance (HOMA-IR), and triglycerides, while changes in BMI were not observed in this sample of chronic patients treated with first- and second-generation antipsychotic and eventually with anticholinergic agents.

Akkermansia muciniphila

*A. muciniphila* is a more effective microbe in weight reduction and metabolic syndrome management [71]. *A. muciniphila*, a mucin-degrading bacteria, belongs to Verrucomicrobia phylum and is commonly present in healthy human microbiota, in a concentration ranging from 3% to 5% [28]. The concentration of *A. muciniphila* is greater in healthy subjects than in those with diabetes, obesity, metabolic syndrome, or chronic inflammatory diseases [15], trials on rodents and humans demonstrate its promising role in the treatment of these pathologies [72].

*A. muciniphila* can increase the gut mucus layer through the production of SCFAs and by increasing the number of goblet cells and promoting claudine 3 and occludin synthesis, thus improving the intestinal barrier and reducing the lipopolysaccharides (LPS) passage in the blood with its pro-inflammatory activity [73,74,16]. *A. muciniphila* produces Amuc_1100, a specific bacterial protein that improves glucose and lipid metabolism and the gut barrier by its direct action on enterocytes and produces P9, a protein that promotes GLP-1 release by intestinal L cells greater than SCFAs, thus contributing to the positive metabolic effects and improving thermogenesis in brown adipose tissue [74-76]. *A. muciniphila* can modulate the intestinal immune system by phosphatidylethanolamine with two branched chains (a15:0-i15:0 PE), produced only by this bacterium and can modulate the cannabinoid system positively influencing inflammation and intestinal barrier function [77,78].

Administration of *A. muciniphila* in obese mice can reduce weight, glucose impairment, insulin resistance, gut permeability, and LPS level, improving metabolic syndrome and non-alcoholic fatty liver disease, low-grade inflammation, and immune cell white fat infiltration [16,79-81]. In 2019 the first RCT was conducted for three months on 40 obese or overweight insulin-resistant human volunteers to evaluate safety, tolerability, metabolic parameters (insulin resistance, circulating lipids, visceral adiposity, and body mass), gut barrier function (plasma lipopolysaccharides), and gut microbiota composition. Administration of pasteurized *A. muciniphila* as a postbiotic has shown a 30% reduction of insulin resistance, a 20% decrease of hepatic markers, a 117% reduction of circulation LPS, while the average weight decrease was 2.27 kg, and a 2.63 cm reduction of waist circumference [17].

Administration of *Akkermansia muciniphila* in olanzapine weight induced obesity in the rodent model

Two groups of female mice, one treated with a high-fat diet for eight weeks to induce obesity and one with a normal chow diet were randomly divided into three groups: one treated with phosphate-buffered saline, one with olanzapine, and the latter with olanzapine and *Akkermansia muciniphila* for 16 weeks, in order to study weight and metabolic changes in the lean and obese group over time [82]. Olanzapine can cause weight gain in normal chow diet-fed mice without affecting food intake probably by decreasing energy expenditure, while obese mice reported no further weight increase, though they showed poor metabolic outcomes over time.

Olanzapine administration exacerbated hyperglycemia in mice and insulin resistance, this effect was time-dependent increasing over the weeks. *A. muciniphila* significantly improved glucose intolerance in both lean and obese mice, reducing mRNA expression levels of glucose-6-phosphatase (G6Pase) and phosphoenolpyruvate carboxykinase (PEPCK), key enzymes in hepatic gluconeogenesis, thus ameliorating olanzapine associated insulin resistance. Though weight loss was non-statistically significant and despite the limitations due to a 12-week period of probiotic administration, it has been shown that *A. muciniphila* can contribute to weight loss over time. *A. muciniphila* alleviates olanzapine-induced systemic inflammation, a hallmark of several metabolic diseases, in both groups highly significantly reducing IL-6 and TNF-α levels. Olanzapine treatment reduced goblet cell numbers in mice, which was reversed by *A. muciniphila* administration, in both groups probiotic administration increased colonic expression of occludin, a tight junction protein implicated in gut barrier function. Obese mice with olanzapine treatment manifested signs of hepatic injury with increases in serum ALT and AST that were significantly reversed by *A. muciniphila* coadministration. No changes were detected in lipid blood dosage, though olanzapine in addition to stimulating lipogenesis is reported to dysregulate lipid metabolism by inhibiting the expression of lipolysis-related genes [83].

One of the limitations of this study is not testing the male mice, potentially introducing a sex bias. Moreover, caution is needed in translating the results obtained from animal models to humans, especially to the clinical population. Though weight loss was non-statistically significant and despite the limitations due to a 12-week period of probiotic administration, it has been shown that *A. muciniphila *can contribute to weight balance over time.

The authors demonstrate that this single-strain probiotic has a pleiotropic mechanism of action intervening on the principal pathways on which olanzapine induces weight and metabolic modifications, as shown, in particular, by mRNA analysis. This study shows the contribution of *A. muciniphila* in treating the insulin resistance and hyperglycemia induced by olanzapine, in decreasing systemic inflammation and improving gut barrier function.

Discussion

The role of probiotics in reducing antipsychotic weight gain is still controversial for the weight loss registered in most of the studies, both on mice models and on humans, was not always statistically significant. One of the main factors limiting concluding this parameter is the great heterogeneity of the human studies, not only for the diverse probiotics that were tested alone or in combination with fibers or vitamin D, but also primarily for patient selection and timing of intervention. Only one RCT was conducted on the first episode of psychosis patients, while in the others, several diverse SGAs were administered to subjects treated for a longer period of time [66-70]. More studies are needed to clarify if the role of probiotics is in preventing weight gain when administered at the beginning of antipsychotic treatment or if they have a role even once the antipsychotic weight gain has been established.

Another limiting factor to evaluate probiotic effectiveness in weight balance is the shortness of the studies, each of the RCTs has a 12-week duration, and longer period researches are recommended because commonly the SGAs are provided as a long-term therapy [66-70]. All RCTs probiotics have been demonstrated to statistically significantly improve hyperglycemia and insulin resistance, this was observed in every stage of SGA treatment both in patients with first episode of psychosis and chronic antipsychotic administration and the results are evident in 12 weeks [66-70].

Although probiotic trials on the psychiatric clinical population are still needed in order to estimate the real efficacy and effectiveness of weight gain and metabolic impairment treatment, several data on probiotic safety and tolerability are available as confirmed in all the RCTs examined, so they can be proposed in an integrated intervention [66-70].

Focusing on olanzapine, one of the SGAs that more frequently causes weight implementation, the role of the diverse probiotics tested in reducing weight gain, has not produced clear evidence yet. In a mice model study, VSL#3 produced a significant weight decrease [45], while *Akkermansia muciniphila* induced a positive but non-significant weight loss at the end of the trial [17] and in three RCTs conducted on a clinical population the diverse probiotic alone or together with fibers evidenced a contribution on weight balance, especially at the beginning of the treatment [67-69].

*A. muciniphila* was administered only in one RCT on mice treated with olanzapine with positive but not significant results on weight loss, and statistically significant improvement of insulin resistance, hyperglycemia, inflammation, liver function, and gut barrier function, showing to intervene on the very parameters altered by olanzapine [17].

## Conclusions

SGAs can be the best choice in the treatment of major psychiatric disorders but it is known that they can induce several side effects, such as weight gain and metabolic disorders. For the clinician, it is important to manage both psychiatric symptom control and metabolic side effects for long-term patient health and survival outcomes. As the SAG weight gain is quickly induced, a corrective treatment should be started early. SGAs modification on gut microbiota is well known in both animal and human studies, consequently, microbiota modulation can be a complementary approach for psychiatric patients with metabolic impairment and weight gain induced by treatment. The role of probiotics in reducing antipsychotic weight gain is still controversial as the weight loss registered in most of the studies was not always statistically significant. The great heterogeneity of human studies for the type of probiotic intervention, patient selection, and timing of intervention limits drawing a clear conclusion on the efficacy and effectiveness of probiotics in reducing antipsychotic-induced weight gain, while all of the studies showed a significant contribution in mitigating the metabolic side effects in each stage of SGAs treatment. In all of the studies, both on mice models and in human ones, probiotic intervention has shown to be safe and well tolerated.

Among microbiota modulation interventions, *A. muciniphila*, which has recently been made available as a postbiotic for human use, has shown a unique metabolic and weight balance profile with emergent evidence attested in human trials with metabolic disorders. This bacteria strain gut concentration is reduced in patients with diabetes, obesity, metabolic syndrome, or chronic inflammatory diseases, and it is reduced in SGAs-treated patients. *A. muciniphila* has shown to act on the same parameters and pathways altered by olanzapine, one of the SGAs that more frequently induces weight gain and metabolic impairment. *A. muciniphila* administered to olanzapine treated mice has demonstrated to significatively improve hyperglycemia, insulin resistance, inflammation parameters, liver, and gut barrier function, and it has shown to induced a positive but non-significant weight loss at the end of the trial. To better understand the role in prevention or treatment of olanzapine-induced weight gain, studies on the psychiatric population are needed, but due to its pleiotropic mechanism of action, proposing integration to *A. muciniphila* to psychiatric patients with a SGAs induced altered metabolic and weight profile could be a rational and safe treatment complement.
